# Environmental Enrichment Attenuates Fentanyl-Seeking Behavior and Protects against Stress-Induced Reinstatement in Both Male and Female Rats

**DOI:** 10.1523/ENEURO.0447-25.2026

**Published:** 2026-04-16

**Authors:** Jessica A. Higginbotham, Joanna J. Dearman, Mateo Pujol, Rachel Teich, Hasan Maqbool, Jose A. Moron

**Affiliations:** ^1^Department of Anesthesiology, Washington University in St. Louis, St. Louis, Missouri 63110; ^2^Pain Center, Washington University in St. Louis, St. Louis, Missouri 63110; ^3^School of Medicine, Washington University in St. Louis, St. Louis, Missouri 63110; ^4^Departments of Neuroscience, Washington University in St. Louis, St. Louis, Missouri 63110; ^5^Psychiatry, Washington University in St. Louis, St. Louis, Missouri 63110

**Keywords:** addiction, environmental enrichment, fentanyl, self-administration, stress

## Abstract

Environmental enrichment (EE) reduces vulnerability to multiple drugs of abuse, yet its impact on fentanyl use and relapse-like behavior remains unclear. Here, we tested whether long-term, nonsocial, object-based EE alters fentanyl self-administration, extinction, and stress-induced reinstatement in male and female rats. Rats were individually housed in either standard nonenriched (NE) conditions or in EE cages containing a rotating set of novel objects beginning at least 3 d prior to self-administration. EE did not impact acquisition of fentanyl self-administration but reduced fentanyl intake during maintenance of self-administration and reduced the persistence of drug-seeking in extinction. Following extinction, yohimbine robustly reinstated drug-seeking behavior in NE rats but reinstatement in EE rats was markedly attenuated, indicating reduced sensitivity to stress-induced relapse triggers. Circulating corticosterone levels were lower in EE rats across the experiment and were positively correlated with reinstatement responding, suggesting that enrichment's protective effects may be mediated in part by reduced hypothalamic-pituitary-adrenal (HPA) axis activation. These findings demonstrate that object-based EE, even in the absence of social enrichment, is sufficient to blunt fentanyl use, facilitate extinction, and constrain stress-induced reinstatement. The results highlight the role of environmental context and stress regulation in fentanyl vulnerability and suggest that enrichment-inspired, nonsocial interventions may offer a scalable strategy to reduce opioid use and relapse risk.

## Significance Statement

This study demonstrates that long-term, object-based environmental enrichment in the absence of social peers is sufficient to reduce fentanyl intake and blunt stress-induced reinstatement in male and female rats. These findings identify a simple, scalable environmental intervention capable of dampening both drug use and vulnerability to relapse triggers. Given the prevalence of individuals exposed to periods of social isolation or with limited access to support, understanding how nonpeer-based enrichment influences maladaptive opioid use and stress reactivity is of timely and translational importance. The ability of enrichment to reduce corticosterone, fentanyl consumption, and drug–cue reactivity highlights the importance of environmental context in shaping opioid misuse liability and implicates novel and practical avenues for relapse prevention.

## Introduction

The current opioid epidemic remains a serious and escalating public health crisis. Since 2013, overdose has been the leading cause of injury death in the United States (CDC/NCHS, 2019). During the COVID-19 pandemic, opioid-related deaths rose sharply, increasing by 32% between 2019 and 2020 (Ghose et al., 2022). Fentanyl accounted for the majority of these fatalities, with a 55% increase observed during the first year of the pandemic (Mattson et al., 2021; Garnett and Miniño, 2024). Beyond drug availability, pandemic-related factors—including social isolation, financial instability, and other stressors—disproportionately affected underserved communities (Chacon et al., 2021; Currie et al., 2021; Fallon et al., 2021; Manchikanti et al., 2021). These environmental stressors likely contributed to increased opioid use and relapse, emphasizing the need to understand how external stimuli shape opioid use, stress system function, and relapse vulnerability.

Stress is a well-established trigger for relapse. Early clinical studies demonstrated that relapse was positively associated with self-reported stressful life events following abstinence (Kosten and George, 2002). These observations were subsequently modeled in animals using the reinstatement paradigm, in which drug-seeking behavior is reinstated after abstinence or extinction by exposure to one of three relapse-inducing stimuli: a subthreshold drug dose, drug-associated cues, or stress (Shaham et al., 2003; Banna et al., 2010; Bossert et al., 2013). A key shared feature among these stimuli is their capacity to activate the hypothalamic pituitary adrenal (HPA) axis, the central stress response system (Armario, 2010; Mantsch et al., 2016; Higginbotham et al., 2021; Greenwald et al., 2024). Accordingly, the stress-induced reinstatement procedure provides a valuable framework for examining neurobiological mechanisms underlying relapse. Traditional stressors in this model include footshock, forced swim, or tail pinch; however, pharmacological stressors such as kappa opioid receptor agonists, corticotropin-releasing factor, and the α2-adrenoceptor antagonist, yohimbine, are now more commonly used (Mantsch et al., 2016). Yohimbine reliably induces HPA axis activation across species and readily instigates opioid-seeking behavior (Banna et al., 2010; Greenwald et al., 2013, 2024). Therefore, yohimbine-mediated stress-induced reinstatement offers a translationally relevant approach for assessing the relationship between stress and relapse.

Environmental factors also have profound effects on drug craving and relapse. Environments associated with past drug use, or those lacking adequate health-related, financial, or educational resources, can provoke drug-seeking behavior in animal models and relapse in humans (Tucker et al., 1991; Kendler et al., 2003; Rhodes et al., 2003; Crombag et al., 2008; Stringfield et al., 2016, 2017; Higginbotham et al., 2021; Razali et al., 2023). Conversely, environments that promote cognitive stimulation, social support, and/or physical activity can reduce drug craving and promote abstinence in both clinical and preclinical settings (Galaj et al., 2020). In rodent models, environmental enrichment (EE) is used to simulate positive and stimulating experiences by increasing the complexity of the living environment (Solinas et al., 2021). Effective and translational EE provides animals with opportunities for voluntary engagement in naturally rewarding, safe activities (Solinas et al., 2021), typically achieved by housing animals with multiple novel objects and at least one social peer, while control conditions typically include social-only housing (peers without objects) or isolated housing (neither peer nor objects; Malone et al., 2022). Numerous studies have reported the therapeutic effects of EE on behavioral measures related to drug abuse vulnerability (Solinas et al., 2021; Malone et al., 2022); however, these group-based enrichment conditions do not clarify whether beneficial effects of EE require both peers and objects or can be conferred by objects alone. This gap is particularly relevant given the increased prevalence of social isolation due to COVID-19 restrictions (Davim et al., 2021) and the growing number of individuals working remotely or in hybrid environments (Fostervold et al., 2024). Therefore, determining the therapeutic potential of enrichment in isolation is of timely importance.

Despite extensive work on EE, it has not been investigated whether object-based enrichment in isolation can reduce maladaptive fentanyl use or protect against stress-induced reinstatement of fentanyl-seeking behavior. Prior research shows that EE can lower baseline corticosterone levels—a physiological correlate of the canonical stress hormone cortisol in humans (Belz et al., 2003)—and reduce reinstatement for both natural and drug rewards (Grimm et al., 2008; Chauvet et al., 2009; Hofford et al., 2017; Imperio et al., 2018; Solinas et al., 2021). However, neither EE nor the stress-induced reinstatement model has been applied in the context of fentanyl. To address these gaps, the current study examined the efficacy of long-term, nonpeer, object-based environmental enrichment in reducing fentanyl use and stress-induced relapse-like behavior in individually housed male and female rats. We further assessed whether EE altered baseline corticosterone concentrations and stress reactivity at key time points throughout the study. In the context of the current fentanyl crisis, these experiments aim to provide novel insight into how long-term enrichment in isolation impacts fentanyl use, reinstatement, and stress reactivity.

## Materials and Methods

### Animals

Male and female wild-type Long–Evans rats (*n* = 37; 225–470 g) were bred in house (*n* = 27) or purchased from Inotiv (*n* = 10). Notably, breeding source did not influence self-administration behavior (RM 2-way ANOVA; Day: *F*_(14,490)_ = 3.620, *p* < 0.0001; Source: *F*_(1,35)_ = 1.742, *p* = 0.1954; Day × Source: *F*_(14,490)_ = 1.247, *p* = 0.2373) or corticosterone concentrations (RM 2-way ANOVA; Time point: *F*_(4,136)_ = 14.04, *p* < 0.0001; Source: *F*_(1,37)_ = 1.286, *p* = 0.2640; Time point × Source: *F*_(4,136)_ = 2.168, *p* = 0.0759) throughout the experiment. All rats were 10–14 weeks old at the start of the experiment. Rats were given *ad libitum* access to water and were placed on a mild food-restricted diet consisting of ∼10% in body weight (g) of PicoLab Rodent Diet 20 per day. Subjects were housed in temperature- and humidity-regulated conditions under a 12 h light/dark cycle (lights on at 6:00 A.M.). Rats were group housed until they underwent intravenous catheterization and were individually housed thereafter. All animal procedures were performed in accordance with Washington University's animal care committee's regulations.

### Intravenous catheterization surgery

To permit intravenous fentanyl self-administration, catheters were constructed in house with polyethylene tubing (∼13 cm, 0.02” ID, 0.037” OD) and were surgically implanted into the right jugular vein as previously described (Higginbotham et al., 2025). The tubing exited between the scapulae and was connected to a single-channel silicone vascular access harness (Instech Labs, VAHR1H/22). Enrofloxacin (8 mg/kg, s.c.) and carprofen (5 mg/kg, s.c.) and a chewable bacon-flavored Rimadyl tablet (2 mg/tablet; Bio-Serv) were administered 0, 24, and 48 h after surgery. Catheter patency was maintained with daily flushing of 0.3 ml sterile saline containing gentamicin (1.33 mg/ml, i.v.) to mitigate infection. Patency was periodically assessed as needed with propofol (0.1 ml, 10 mg/ml, i.v.), and rats with a loss of patency at time point throughout self-administration were excluded from the study. Following catheterization, rats were randomly assigned to individually housed conditions with or without enrichment (see below, Housing conditions) for the duration of the study.

### Housing conditions

**Figure 1.**
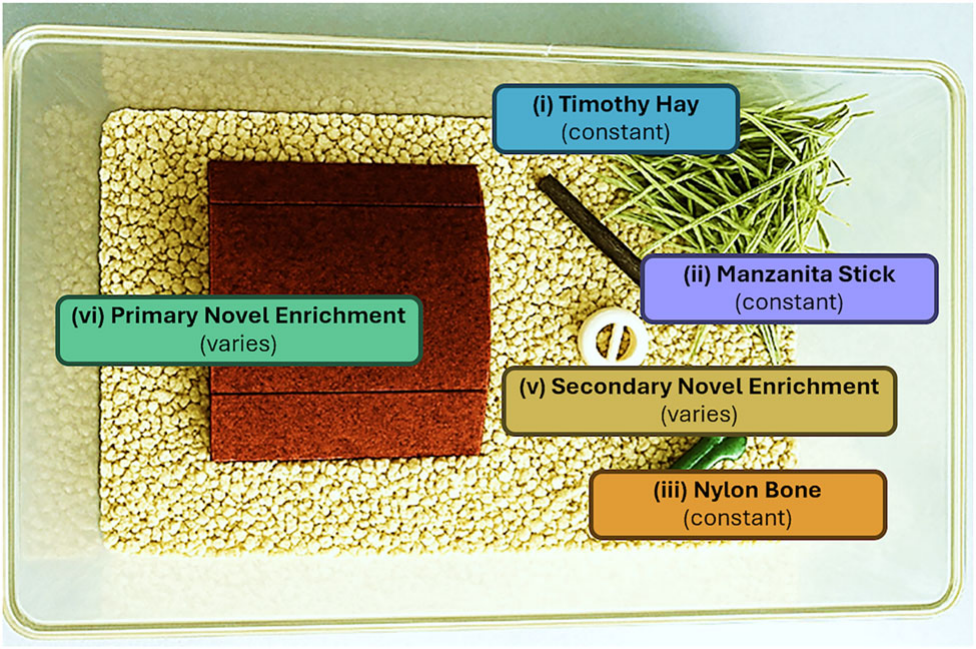
Example of environmentally enriched (EE) conditions consisting of five distinct stimuli (see Table 1 for details).

Rats were assigned to either nonenriched (NE) or environmentally enriched (EE) single-housing conditions for the duration of the experiment (Fig. 1, Table 1), starting 3–5 d before the first day of fentanyl self-administration and continuing through the final reinstatement test. All subjects were individually housed. In both NE and EE conditions, rats were housed in a standard durable, plexiglass cage with corn cob bedding, a stainless steel chow and water rack, and a plastic, filtered cage top. NE conditions had no additional components. EE conditions contained five distinct enrichment items: (1) hand-selected foraging grass (Bio-Serv) (2) manzanita gnawing sticks (Bio-Serv), (3) nylon bones (Bio-Serv), (4) a primary novel enrichment item, and (5) a secondary enrichment item. Primary and secondary enrichment items were obtained from the Fisher Scientific enrichment catalog or fabricated using a 3D printer(Bambu/P1P) with lab grade polylactic acid (PLA) filament (CARBON; Fig. 1). Primary items were categorized as one of the following functions: shelter (e.g., tunnel, hut), texture (e.g., dumbbell, rubber chew), or interactive (e.g., grass woven swing, stainless steel rattles). Secondary enrichment items consisted of a small unsophisticated object (e.g., kong toy, ping pong ball). Novel primary and secondary enrichment items were introduced during each cage change which occurred once per week independent of housing conditions (Table 1).

**Table 1.** Environmental enrichment

Item	Use	Type	Proposed benefit	Source (example)
i	Constant; replaced each week	Timothy Hay	Encourages natural nesting and foraging behavior, aids in dental health	Bio-Serv #S1012
ii	Constant; replaced each week	Manzanita Sticks	Promotes gnawing and aids in dental health, serves as perches or manipulanda	Bio-Serv #W0016
iii	Constant; replaced each week	Nylon Bones	Promotes gnawing behavior and aids in dental health	Bio-Serv #K3580
iv	Primary Novel Enrichment; type changes each week	Shelter Type	Enclosed space adds security, encourages nesting, reduces anxiety	Bio-Serv #K3365 (Rat-Hut); #K3325 (Rat-Tunnel); #K3245 (Rat Retreat)
		Texture Type	Promotes exploratory behavior; encourages forelimb use, reduces boredom	Bio-Serv #K322 (Dumbells); #K3289 (DNA Flexor); #K3290 (Football); #K341 (Jump'n Jacks)
		Interactive Type	Encourages movement; provides mental stimulation; engages curiosity through object novelty and portability	Fisher Scientific #14-727-066 (rattles); #14-727-032 (ball and chain); Rat foraging Toy 3d Print File
v	Secondary Novel; similar type changed each week	Nonspecific unsophisticated	introduces novelty; serves as additional manipulanda with portability	Airless Golfball 3D Print File Rattling Ball 3D print file

### Drugs

Fentanyl stock solution (50 μg/ml) was diluted with a sterile saline solution to obtain the appropriate dose based on body weight (5 or 2 µg/kg/100 μl infusion). Yohimbine (ThermoFisher) was dissolved in a 1:1 solution of sterile deionized (DI) water and 0.9% saline using sonication, yielding a final concentration of 2.5 mg/ml.

### Fentanyl self-administration, extinction, and reinstatement

Rats were maintained in their assigned housing conditions (implemented 3–5 d before starting training) throughout the duration of the experiment. The fentanyl self-administration, extinction, and reinstatement procedure was conducted as previously described (Higginbotham et al., 2025). All procedures were performed in a standard operant chamber (Med Associates, RRID: SCR_021938) equipped with two retractable levers, a cue light above each lever, a stainless steel grid flooring, and a house light. Rats were naive to the apparatus and instrumental training prior to the start of self-administration. For fentanyl self-administration (SA) sessions, rats were placed in operant chambers and tethered to the infusion line. Rats underwent 15 2 h SA training sessions under a fixed-ratio 1 (FR1) reinforcement schedule. Responses on a designated active lever resulted in illumination of the light cue above the active lever (5 s) and the programmed delivery of a single fentanyl infusion (100 μl, 5.24 s) followed by a 20 s time-out period during which additional active lever responses did not result in added reinforcement. Responses on the inactive lever were recorded but had no program consequences. Rats were allowed to self-administer a moderate dose of fentanyl dissolved in sterile saline during the first 5 sessions of Acquisition (5 μg/kg/infusion, i.v.) and had access to a lower dose the remaining 10 sessions during Maintenance (2 μg/kg/infusion, i.v.) as previously described (Higginbotham et al., 2025).

**Figure 2.**
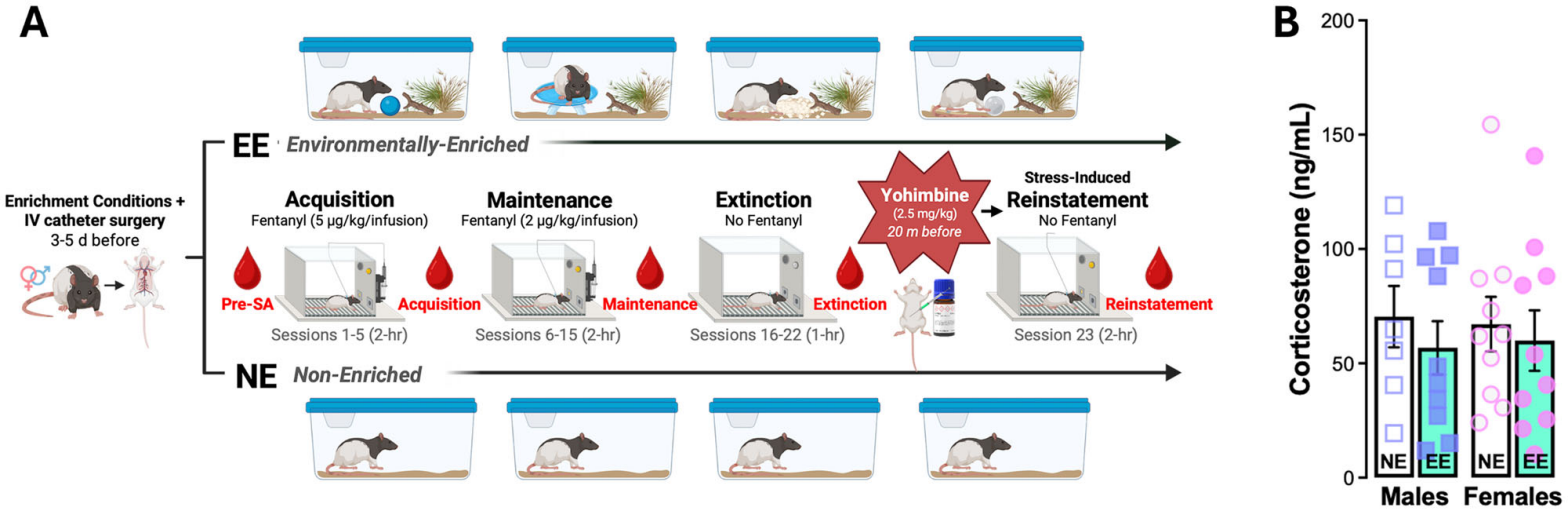
Experimental timeline and baseline corticosterone concentrations. ***A***, Male and female Long–Evans rats received intravenous (IV) jugular catheters 3–5 d prior to the first day of fentanyl self-administration. After surgery, rats were randomly assigned to be individually housed with environmental enrichment (EE) or no enrichment (NE) which were maintained over the course of the experiments. Acquisition of fentanyl self-administration occurred over five 2 h sessions where responses on an active lever triggered a light cue illumination and delivery of a fentanyl infusion (5 µg/kg, i.v.). Subsequent maintenance sessions (6–15) occurred in the same manner except with a lower fentanyl dose of 2 µg/kg, i.v. Upon completing the 15 sessions of fentanyl self-administration, rats underwent seven 1 h sessions of extinction training where responses on the active and inactive lever were recorded but had no programmed consequences. The next day, rats received yohimbine (2.5 mg/kg, i.p.) 20 min before the 2 h reinstatement test during which conditions were identical to those during self-administration except fentanyl reinforcement was withheld. Tail vein blood samples were obtained at multiple time points throughout the study (red): 1 d before starting fentanyl self-administration (Pre-SA), after sessions 3 or 4 (acquisition), after one of sessions 8–12 (maintenance), after session 21 or 22 (extinction), and trunk blood was obtained immediately after the final test (reinstatement). ***B***, Prior to starting self-administration (after 3–5 d of NE or EE housing; Pre-SA), there were no differences between males and females with NE or EE in corticosterone concentrations. *N* = 7–10/group; females depicted as circles, and males depicted as squares. Full statistical reporting available in Extended Data Figure 2-1.

Upon completing 15 SA sessions, rats underwent extinction training which took place over the course of seven 1 h sessions in the same operant chamber as SA except that rats were not tethered to the infusion line, and no associated cues or rewards were administered following active and inactive lever responses. On the last day of extinction training, rats were injected with saline (1 ml/kg, i.p.) 20 min before the start of session to habituate them to the injection procedure performed during the subsequent reinstatement session (see behavioral timeline in Fig. 2*A*).

10.1523/ENEURO.0447-25.2026.f2-1 Figure 2-1 Table with statistical reporting for Figure 2. Download Figure 2-1, DOCX file.

For the final test of stress-induced reinstatement, rats received the pharmacological stressor, yohimbine (2.5, mg/kg, i.p.), 20 min before the start of the test. Reinstatement took place in the same operant apparatus as SA and extinction. During the 2 h test, conditions were identical to those present during SA training except that fentanyl reinforcement was withheld. Responses on the active lever were used as an indicator of drug-seeking behavior. Immediately following completion of the 2 h test, rats were deeply anesthetized and decapitated, and trunk blood was obtained in lieu of tail blood (see below) to assess corticosterone concentrations during reinstatement.

### Tail blood collection

Blood samples were obtained between 11:00 A.M. and 4:00 P.M. upon completing behavioral sessions at five time points throughout the experiment (Fig. 2*A*): (1) 1 d prior to the first fentanyl SA session to establish baseline corticosterone levels (Pre-SA; i.e., 3–5 d after housing conditions were implemented), (2) after one of the first five fentanyl SA sessions (Acquisition), (3) after one of SA sessions 8–12 (Maintenance), (4) once after extinction session 6 or 7 (Extinction), and (5) after administration of yohimbine and the reinstatement test (Reinstatement). All blood samples (except Reinstatement) were obtained vial tail nick of the lateral tail vein as previously reported (Higginbotham et al., 2021). Briefly, rats were swaddled, and the distal end of the rat's tail was secured while a sterile razor was used to nick <1 cm parallel to the lateral tail vein. The tail was milked from the proximal to the distal end using the thumb and index finger, and blood samples were collected in serum separating tubes. Samples coagulated at room temperature for 1 h before they were centrifuged for 15 min at 4°C at 13,000 rpm. The separated serum was decanted and aliquots of 25–50 μl were prepared and stored at −80°C until assayed.

### Corticosterone analyses

Serum corticosterone concentrations were determined using a competitive enzyme immunoassay ELISA kit (Bio-Techne; sensitivity, 0.047 ng/ml; range, 0.103–25 ng/ml) according to the assay instructions. Briefly, samples were thawed on ice, pretreated, and diluted 8× with calibrator diluent. Duplicates were run from each sample and standard on multiple plates. The pretreated 96-well plate was incubated with a polyclonal antibody specific for corticosterone, and a substrate solution was added to the wells to determine the bound enzyme activity determined by the absorbance. Plates were read at 450 nm within 15 min of development. Duplicate readings for each standard and sample were averaged, and the optical density (OD) of nonspecific binding (NSB) was subtracted. A standard curve for each plate was generated using a four parameter logistic (4-PL) curve (*R*^2^ = 0.979–0.991). Then corticosterone concentrations were determined based on the interpolated value from the standard curve and multiplied by the dilution factor. Values outside the range of detectability were excluded from the analysis.

### Statistical analysis

All statistical analyses were conducted using GraphPad Prism. Data were assessed for normality and sphericity; Greenhouse–Geisser corrections were applied where necessary. Behavioral data from self-administration, extinction, and reinstatement phases were analyzed using repeated-measures three-way ANOVAs with session as the within-subjects factor and housing condition [environmental enrichment (EE) vs nonenrichment (NE)] and sex (male vs female) as between-subjects factors. When there were no sex effects in these analyses, statistical comparisons were carried out with repeated-measures two-way ANOVAs using enrichment as the between-subjects factor (Extended Data Figs. 2-1, 4-1, 5-1, 6-1). When three-way interactions were observed in the absence of any two-way interactions or main effects (Extended Data Fig. 3-1), repeated-measures two-way ANOVAs were performed for each factor. Two-way ANOVAs were used to assess corticosterone concentrations with housing condition and sex as between-subject factors. The relationship between corticosterone concentrations and lever pressing was assessed using simple linear regressions. For all ANOVAs, Tukey's and Sidak's multiple-comparisons tests were used for post hoc comparisons when interactions were statistically significant. Comprehensive reporting of all statistical analyses performed are in Extended Data Figures 2-1, 3-1, 4-1, 5-1, 6-1. Statistical significance was set at *α* = 0.05 for all tests. Data are presented as mean ± SEM.

## Results

### Prior to fentanyl self-administration, environmental enrichment in isolation does not alter baseline corticosterone concentrations

Following intravenous catheterization surgery and postoperative care, subjects were acclimated to their nonenriched (NE) or environmentally enriched (EE) isolated housing conditions (Fig. 1) for at least 3 d before tail blood samples were obtained to assess baseline differences in corticosterone concentrations. Once implemented, housing conditions were maintained for the duration of the experiment. After 3–5 d in isolated EE or NE conditions and prior to starting fentanyl self-administration (Pre-SA), there were no differences in corticosterone concentrations between EE and NE groups (Fig. 2*B*; Extended Data Fig. 2-1) indicating that short-term EE (3–5 d) in the absence of fentanyl exposure does not impact baseline HPA axis activation.

### Environmentally enriched rats readily acquire fentanyl self-administration

**Figure 3.**
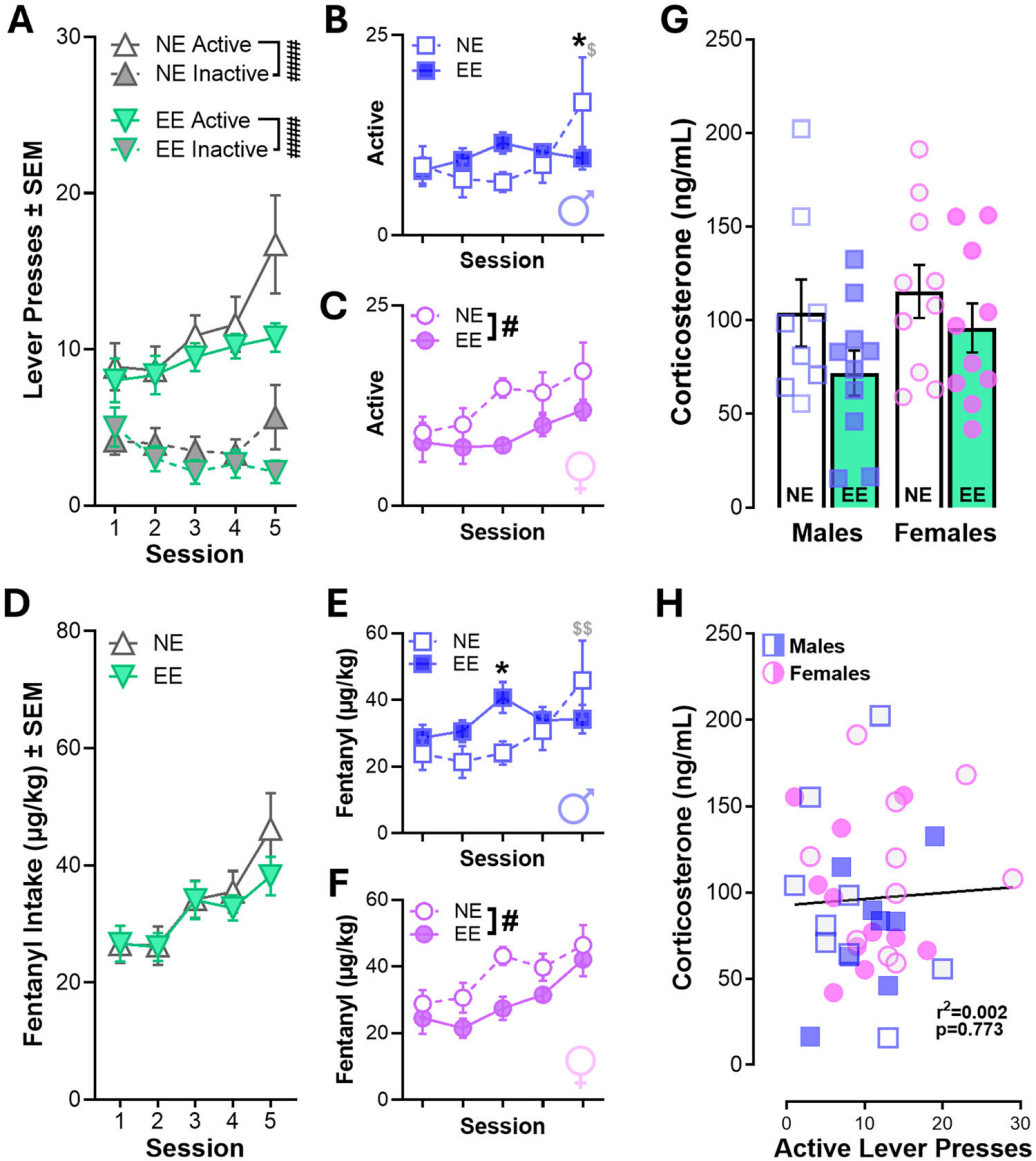
Acquisition of fentanyl self-administration in enriched and nonenriched housing conditions. ***A***, Both environmentally enriched (EE, *n* = 18) and nonenriched (NE = 19) groups acquire fentanyl self-administration and discriminate between the active and inactive lever. ***B***, In males, NE rats responded more on the active lever on the last day of acquisition than EE rats and relative to the first day of acquisition (time effects not shown). ***C***, In females, NE rats exhibited higher lever pressing than EE rats throughout acquisition. ***D***, Fentanyl intake was similar between NE and EE groups, increasing over time. These similarities were due to differences in the trajectory of responding between sexes. ***E***, In males, EE rats self-administered more fentanyl on the third day of acquisition, but only NE rats increased intake by end of the 5 d of training. ***F***, In females, fentanyl intake was overall higher in NE rats than EE rats. ***G***, Corticosterone concentrations in males and females with NE or EE housing with a trending reduction in EE groups (*p* = 0.06). ***H***, Corticosterone levels were not related to responding during training. RM three-way ANOVAs (Extended Data Fig. 3-1), RM two-way ANOVAs (***A***–***F***), ordinary two-way ANOVA (***G***), and simple linear regression (***H***). *N* = 8–10/group; females depicted as circles, males depicted as squares, and both depicted as triangles. Symbols indicate **^#^**main effects or Tukey's post hoc comparisons (performed after 2-way interaction) showing *between-group differences or **^$^**within-group differences relative to the first session (*p < 0.05, **p < 0.01, ***p < 0.001, ****p < 0.0001). Full statistical reporting available in Extended Data Figure 3-1.

Rats received no prior training or exposure to the operant chambers prior to starting fentanyl self-administration. During acquisition, rats underwent five 2 h sessions of fentanyl self-administration (5 µg/kg/infusion; Fig. 3*A*). Housing enrichment conditions did not impact the ability of rats to discriminate between the active and inactive lever and both groups readily acquired fentanyl self-administration regardless of sex and increased active lever responding over time (Fig. 3*A*; Extended Data Fig. 3-1). Evaluating active lever responding during acquisition revealed a three-way interaction between session, housing, and sex (*p* = 0.04) in the absence of other enrichment or sex effects (Extended Data Fig. 3-1). In males, there was a session × enrichment interaction that revealed differences between EE and NE males on the last day of acquisition. NE rats responded more on the last day of acquisition than EE groups and more than the first day of training (Fig. 3*B*; Extended Data Fig. 3-1). In females, EE rats had fewer active lever responses than NE rats across acquisition (Fig. 3*C*; Extended Data Fig. 3-1). Likewise, analysis of fentanyl intake during acquisition produced a three-way interaction, but there were no effects of enrichment when sexes were collapsed (Fig. 3*D*; Extended Data Fig. 3-1). In males, EE rats showed similar intake across the five sessions of acquisition but had higher intake than NE rats during the third session. Despite this, NE rats had higher intake on the last day of acquisition relative to the first day (when both groups were similar; Fig. 3*E*; Extended Data Fig. 3-1). Similar to active lever responding, fentanyl intake in females was overall higher in rats with NE compared with EE (Fig. 3*F*; Extended Data Fig. 3-1). Interestingly, there were no differences in corticosterone concentrations related to sex or housing (Fig. 3*G*), and these levels were similarly unrelated to lever responding during acquisition (Fig. 3*H*; Extended Data Fig. 3-1). Given that differences between EE and NE behavior varied between sex without impacting discrimination, these findings suggest that environmental enrichment does not alter the ability to acquire drug–cue associations but may instead reduce the salience of drug cues or drug reinforcement in a sex-specific manner.

10.1523/ENEURO.0447-25.2026.f3-1 Figure 3-1 Table with statistical reporting for Figure 3. Download Figure 3-1, DOCX file.

### Environmentally enriched rats self-Administer less fentanyl during maintenance

**Figure 4.**
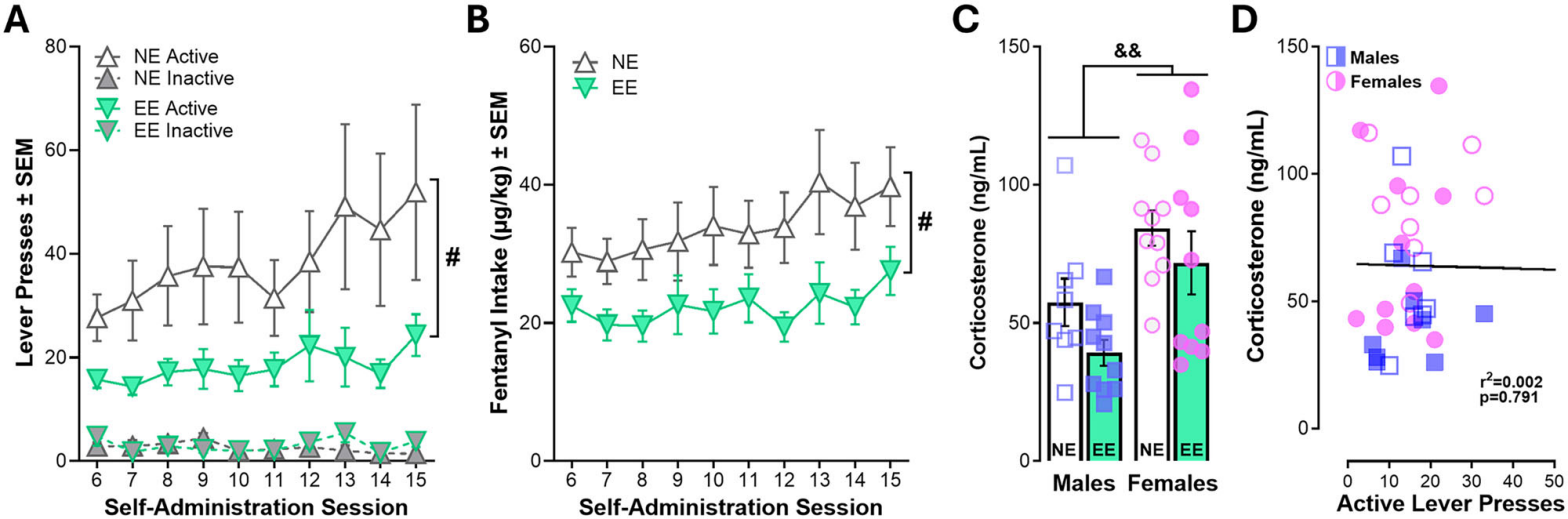
Maintenance of fentanyl self-administration is lower in rats with environmental enrichment. ***A***, Active and inactive lever pressing during maintenance of fentanyl self-administration (sessions 6–15). Independent of sex, EE rats respond less on the active lever across the 10 maintenance sessions leading to (***B***) attenuated fentanyl intake. ***C***, Corticosterone concentrations in females are higher than males. ***D***, Corticosterone levels were not related to responding during training. RM three-way ANOVA (Extended Data Fig. 4-1), RM and ordinary two-way ANOVAs (***A***–***C***), and simple linear regression (***D***). *N* = 8–10/group; females depicted as circles, males depicted as squares, and both depicted as triangles. Symbols indicate main effects of **^#^**housing (*p* < 0.05) or ^&&^sex (*p* < 0.01). Full statistical reporting available in Extended Data Figure 4-1.

Maintenance of fentanyl self-administration continued in ten subsequent 2 h sessions, except that the dose of fentanyl was lower (2 µg/kg/infusion). Interestingly, the sex effects previously observed during acquisition were absent maintenance. Independent of sex, both groups continued to preferentially respond on the active lever, but NE rats responded more on the fentanyl-paired lever than EE rats across the 10 sessions (Fig. 4*A*; Extended Data Fig. 4-1). Similarly, fentanyl intake was, overall, higher in NE rats relative to EE (Fig. 4*B*), demonstrating that EE rats have lower fentanyl intake. Despite the lack of impact of sex during maintenance, we observed a sex effect on corticosterone concentrations examined following maintenance sessions, in that females were higher than males, but any effects of EE did not reach statistical significance (*p* = 0.06; Fig. 4*C*). Nevertheless, corticosterone levels were unrelated to responding during the maintenance session for which samples were obtained (Fig. 4*D*; Extended Data Fig. 4-1).

10.1523/ENEURO.0447-25.2026.f4-1 Figure 4-1 Table with statistical reporting for Figure 4. Download Figure 4-1, DOCX file.

### Environmentally enriched rats respond less during extinction

**Figure 5.**
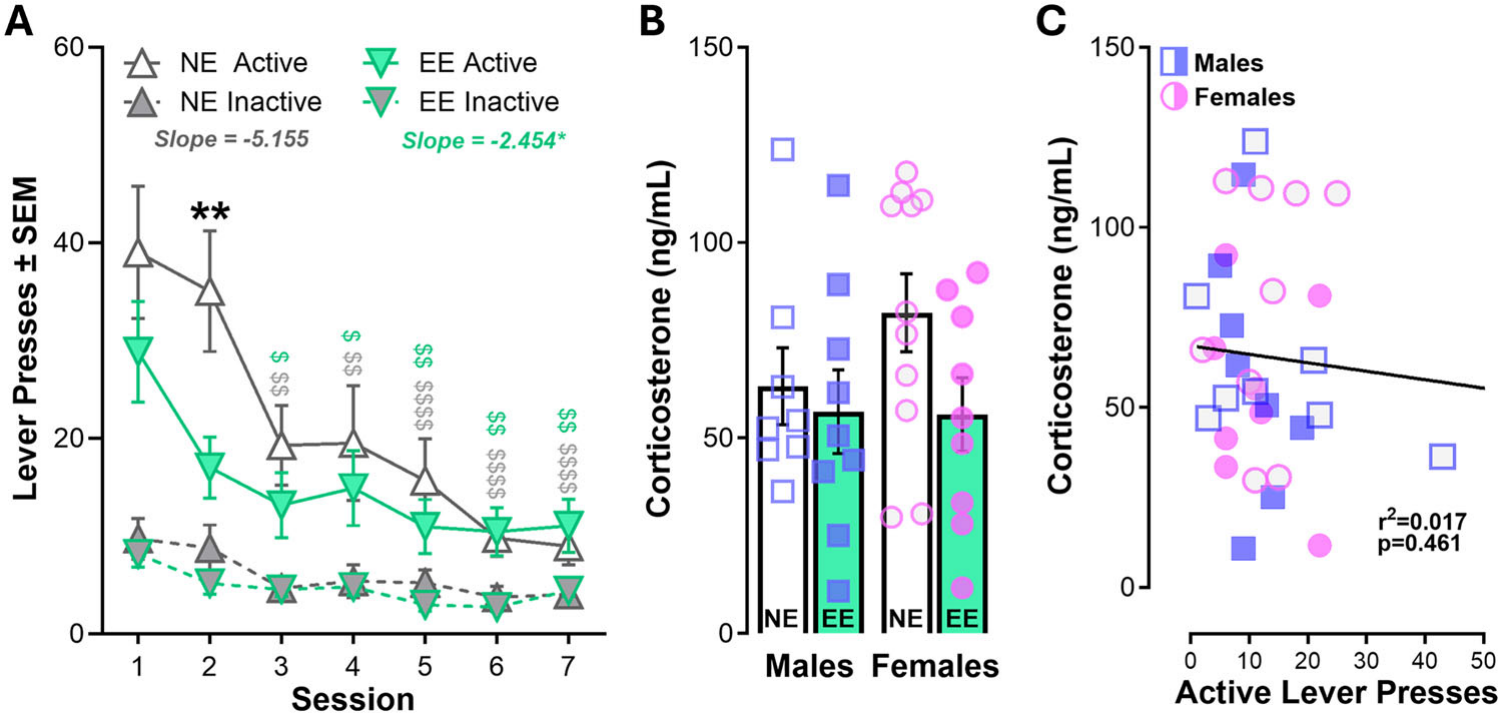
Extinction of fentanyl self-administration. ***A***, Active and inactive lever pressing during extinction (when fentanyl and cues are absent). Independent of sex, NE rats respond more than EE rats on Day 2 of extinction, but both groups reduce responding over time (time effects not shown). Simple linear regression of active lever responding during extinction revealed significant differences in the slope between NE and EE rats. ***B***, Corticosterone concentrations in NE and EE males and females were not significantly different (***C***) or were they related to responding during training. RM three-way ANOVA (Extended Data Fig. 5-1), RM and ordinary two-way ANOVAs (***A***, ***B***), and simple linear regression (***C***). *N* = 18–19/group (NE/EE); females depicted as circles, males depicted as squares, and both depicted as triangles. Symbols indicate post hoc comparisons (performed after 2-way interaction) showing *between-group differences (*p* < 0.01) and ^$^within-group differences relative to the first extinction session (*p < 0.05, **p < 0.01, ***p < 0.001, ****p < 0.0001). Full statistical reporting available in Extended Data Figure 5-1.

After completing acquisition and maintenance of fentanyl self-administration, rats underwent seven 1 h sessions of extinction training where fentanyl and cue reinforcement were withheld. Independent of housing conditions or sex, rats reduced their responding on the active lever over the seven days of training (Fig. 5*A*). NE rats continued to exhibit high rates of responding during the first two sessions compared with subsequent extinction sessions while EE rats only showed higher drug-seeking on Day 1 (Extended Data Fig. 5-1). Analysis of active lever responding across the 7 d revealed significant differences in the rate of extinction between NE and EE rats, indicating that NE rats show more drug-seeking behavior at earlier time points. This suggests that EE reduces the persistence of drug-seeking in the absence of drug-related cues. Notably, these behavioral differences did not translate to differences in corticosterone concentrations (Fig. 5*B*,*C*).

10.1523/ENEURO.0447-25.2026.f5-1 Figure 5-1 Table with statistical reporting for Figure 5. Download Figure 5-1, DOCX file.

### Environmentally enriched rats exhibit attenuated reinstatement and sex-specific alterations in stress reactivity

**Figure 6.**
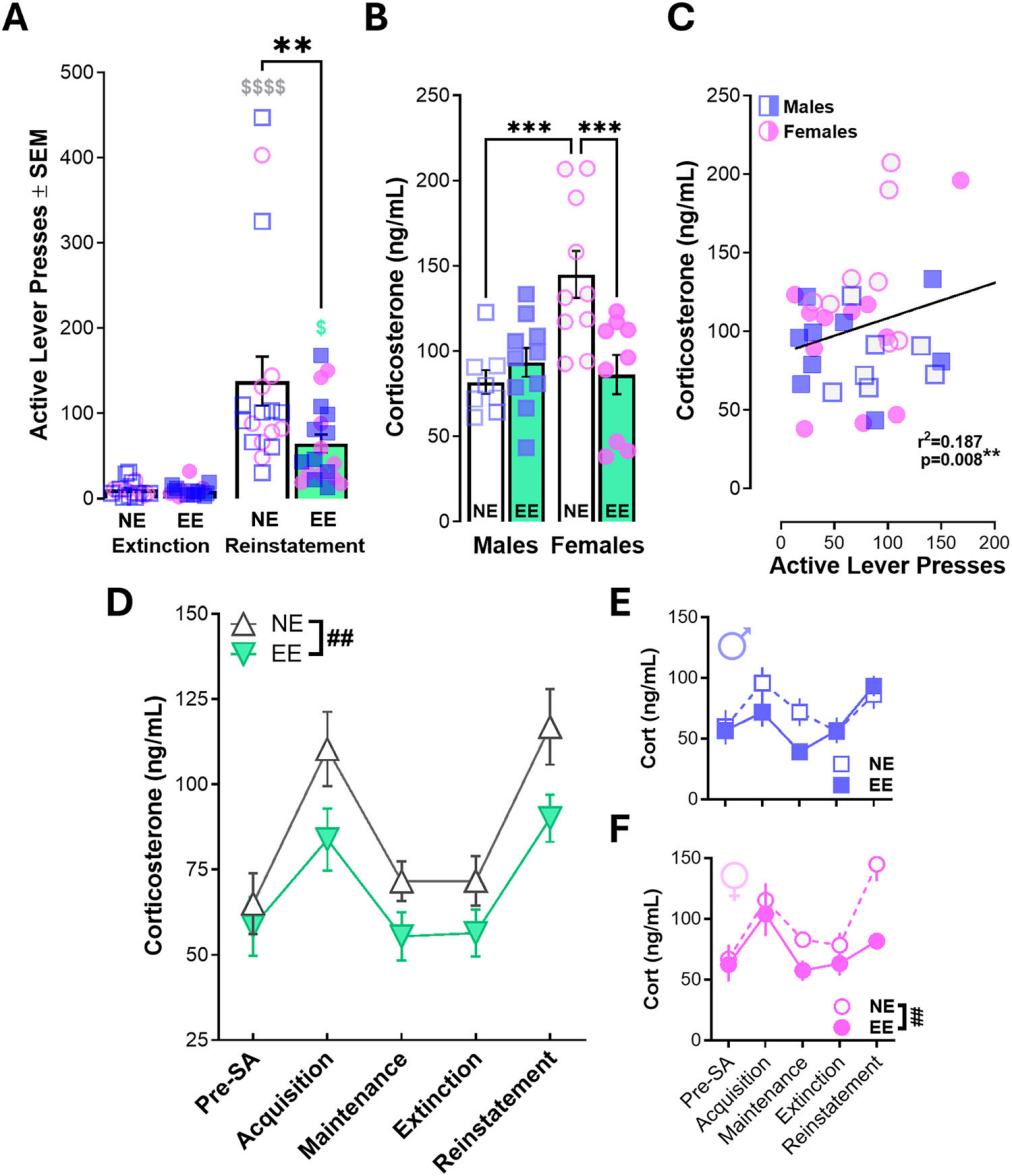
Stress-induced reinstatement of fentanyl-seeking behavior is associated with corticosterone levels and attenuated in environmentally enriched rats. ***A***, Yohimbine (2.5 mg/kg) was administered 20 min before the 2 h reinstatement test. Both groups reinstated based on an increase in active lever pressing during reinstatement relative to extinction. Reinstatement was lower in rats with EE compared with NE independent of sex. ***B***, Corticosterone concentrations after reinstatement were higher in NE females compared with NE males or EE females. ***C***, Corticosterone levels were positively associated with active lever responding during the reinstatement test (*simple linear regression). ***D***, EE rats have lower corticosterone levels than NE rats, but (***E***) these differences were not observed in males. Rather, (***F***) EE reduced overall corticosterone levels specifically in females. RM three-way ANOVA (Extended Data Fig. 6-1), RM two-way ANOVA (***A***, ***D–F***), ordinary two-way ANOVA (***B***), and simple linear regression (***C***). Symbols denote **^#^**main effects of housing or post hoc comparisons (after 2-way interaction) showing *between-group differences or ^$^within-group differences relative to extinction. *N* = 7–10/group; females depicted as circles, males depicted as squares, and both depicted as triangles (*p < 0.05, **p < 0.01, ***p < 0.001, ****p < 0.0001). Full statistical reporting available in Extended Data Figure 6-1.

The next day after the last extinction session, rats underwent a test of stress-induced reinstatement precipitated by Yohimbine (2.5 mg/kg, i.p.) administered 20 min before the session. During the test, rats were tethered to the infusion line but fentanyl reinforcement was withheld. Active lever responses during the test were used as a proxy of drug-seeking behavior. Independent of housing conditions and sex, rats reinstated fentanyl-seeking behavior as indicated by a significant increase in active lever responses relative to the previous day in extinction (Fig. 6*A*). However, the magnitude of reinstatement was greater in NE rats relative to EE rats, demonstrating that EE rats have blunted stress-induced reinstatement. Interestingly, NE females had higher corticosterone responses than EE females or NE males after reinstatement (Fig. 6*B*). However, corticosterone levels were positively associated with active lever pressing during the test session independent of sex (Fig. 6*C*), suggesting the enrichment effects on behavior were related, in part, to stress levels instigated by yohimbine. Assessment of group corticosterone levels across the experimental timeline revealed main effects of enrichment and sex (Fig. 6*D*; Extended Data Fig. 6-1). Upon further investigation, male corticosterone levels were not significantly impacted by enriched housing conditions (Fig. 6*E*; Extended Data Fig. 6-1). Instead, the lower corticosterone concentrations observed in EE rats across the experiment was largely driven by females (Fig. 6*F*; Extended Data Fig. 6-1). As such, females may have greater sensitivity to different housing conditions. Nevertheless, these findings demonstrate the efficacy of object-based enrichment in isolation in reducing fentanyl use and stress-induced drug craving.

10.1523/ENEURO.0447-25.2026.f6-1 Figure 6-1 Table with statistical reporting for Figure 6. Download Figure 6-1, DOCX file.

## Discussion

The current study demonstrates that long-term, nonsocial, object-based EE attenuates fentanyl self-administration and is associated with reduced stress reactivity and stress-induced drug-seeking behavior in both male and female rats. These findings indicate that enrichment delivered in the absence of social peers is sufficient to blunt maladaptive opioid use and stress-induced relapse-like behavior. These add to a substantial body of evidence indicating that environmental enrichment can mitigate addiction-like behaviors across drug classes (Chauvet et al., 2009; Solinas et al., 2009; Alvers et al., 2012) and importantly, are the first to show such effects in the context of fentanyl—currently the most lethal contributor to overdose mortality.

### Environmentally enriched rats show attenuated fentanyl self-administration and drug-seeking behavior

Consistent with prior work showing that EE decreases self-administration of psychostimulants, opioids, and alcohol (Green et al., 2002; Stairs and Bardo, 2009; Malone et al., 2022), we found that EE suppressed fentanyl self-administration during maintenance. In particular, housing conditions impacted the trajectory of acquisition of fentanyl self-administration in a sex-specific manner (Fig. 3*A*). In males, NE rats increased their fentanyl responding after 4 d of training, while EE rats exhibited stable responding across the five acquisition sessions. NE females, however, showed elevated responding throughout acquisition compared with EE females (Fig. 3). Interestingly, despite these sex differences during acquisition, the EE rats maintained lower rates of fentanyl self-administration over the subsequent ten maintenance sessions independent of sex (Fig. 4). Importantly, housing conditions did not influence the ability of rats to acquire fentanyl self-administration (Fig. 3) similar to previous findings with amphetamine, cocaine, and nicotine (Fowler et al., 1993; Stairs and Bardo, 2009; Gipson et al., 2011; Malone et al., 2022). The difference in the trajectory suggests that EE may reduce the reinforcing efficacy or incentive salience of fentanyl or fentanyl-associated cues (Thiel et al., 2012). Previous studies have proposed several non-mutually exclusive mechanisms underlying this effect, including increased engagement with alternative nondrug rewards (Cain et al., 2006; Solinas et al., 2010; Rodríguez-Ortega et al., 2018), altered mesocortical dopamine function (Neugebauer et al., 2004; Del Arco et al., 2007; Segovia et al., 2010), and enhanced synaptic plasticity within prefrontal corticostriatal circuits (Nithianantharajah and Hannan, 2006; Gomez et al., 2015).

Our design implements enrichment as object-based novelty and interaction rather than social complexity, a distinction that has been rarely addressed in past research. The fact that enrichment without social peer interaction was sufficient to suppress fentanyl seeking implies that physical and cognitive stimulation—rather than social buffering alone—can shift the valuation of drug reinforcers. Several lines of evidence confer the detrimental effects of social isolation (Christie, 2021) and the benefits of environmental enrichment on maladaptive drug use (Solinas et al., 2010). However, the latter is largely based on effects derived from both peer- and object-based enrichment and therefore fail to discern the efficacy of object-based enrichment independently. Notably, one study examined long- and short-access cocaine self-administration in rats housed in (1) social conditions with object enrichment, (2) social conditions without objects, (3) isolated conditions with object enrichment, and (4) isolated conditions (Gipson et al., 2011). Although not directly compared, the authors showed that rats in isolated conditions with object enrichment self-administered cocaine at levels similar to those with social and object enrichment (Gipson et al., 2011). Similarly, a separate study found that pair-housed rats with EE exhibited less cocaine-seeking behavior in extinction than pair-housed rats without EE (Thiel et al., 2009). Based on these in the context of the current findings, the therapeutic potential of enrichment on addictive behaviors may rely more on object-based novelty and interaction. This notion is particularly relevant for individuals facing isolation due to pandemic-related disruptions or remote work environments.

### Environmentally enriched rats show reduced persistence of drug-seeking during extinction

Although both NE and EE groups extinguished responding across sessions, NE rats exhibited greater persistence in drug-seeking during early extinction sessions (Fig. 5). This aligns with the idea that enrichment enhances cognitive flexibility and accelerates extinction learning (Branchi et al., 2006; Grimm et al., 2008; Thiel et al., 2009). Reduced perseveration suggests that EE rats adapt more readily to the absence of previously learned reinforcement contingencies. Mechanistically, enrichment has been shown to enhance prefrontal cortical function, increase dendritic branching, and upregulate plasticity markers, like BDNF (Ickes et al., 2000; Gomez et al., 2015; Leger et al., 2015). These neuroadaptations may facilitate inhibitory control over drug-seeking responses when drug-related contingencies change.

### Environmentally enriched rats have attenuated stress-induced reinstatement

Strikingly, EE blunted yohimbine-induced reinstatement of fentanyl seeking (Fig. 6). Stress is a primary driver of relapse in humans (Sinha, 2007), and yohimbine-induced reinstatement robustly models stress-precipitated craving via engagement of the HPA axis (Shepard et al., 2004). Our results showing that NE rats exhibit stronger reinstatement than EE rats independent of sex indicate that enrichment modulates vulnerability to stress-triggered relapse-like behavior. Corticosterone analyses further support a stress buffering role for enrichment, but our findings suggest this role may be sex-specific. Females had lower corticosterone concentrations after reinstatement compared with female NE rats, while similar effects were not observed in males. Given that EE females had lower overall corticosterone concentrations throughout the experiment (Fig. 6*F*), females may be more sensitive to deprived housing conditions. While this sex difference in sensitivity did not translate to differences in drug seeking or drug taking, the impact of housing on females may more broadly impact stress buffering systems. This is consistent with past findings that long-term EE lowers basal glucocorticoid levels and dampens physiological stress reactivity (Belz et al., 2003). Importantly, corticosterone was positively correlated with reinstatement responding, suggesting that enriched environments reduce relapse-like behavior, in part, by constraining stress system activation. This interpretation aligns with studies showing that EE reduces anxiety-like behavior, modifies CRF and glucocorticoid receptor expression, and enhances stress coping strategies (Benaroya-Milshtein et al., 2004; Veena et al., 2009).

### Enrichment effects in social isolation: implications and mechanisms

By using individually housed rats in both NE and EE conditions, the current study isolates object-based enrichment effects from potential confounds of social interaction. Surprisingly, few studies have systematically separated these components. Our data show that the therapeutic benefits of enrichment persist even in the absence of social peers, suggesting that cognitive, sensory, and motor engagement alone deliver sufficient buffering against maladaptive opioid use and stress-induced drug craving. This distinction is critical given the increasing social isolation across populations and the need for accessible, nonsocial forms of environmental novelty and stimulation. The protective effects of object-only enrichment may arise from engagement with naturalistic behaviors (gnawing, exploring, manipulating), which can reduce boredom, anxiety, and passivity—factors associated with drug abuse vulnerability (Malone et al., 2022). Moreover, we observed no sex differences in fentanyl self-administration, extinction, or reinstatement. While some studies report female-specific vulnerabilities to opioid and psychostimulant reinforcement (Becker and Koob, 2016), others find minimal sex differences when access, dosing, and stress variables and tightly controlled (Mavrikaki et al., 2017; Townsend, 2021). The absence of sex effects in the current study suggests that the protective impact of object-based EE generalizes across sexes in this paradigm.

### Limitations and future directions

Several limitations warrant consideration. First, although EE reduced reinstatement triggered by yohimbine, it remains unknown whether similar protective effects would extend to cue-induced, drug-primed, or alternative stressor-induced reinstatement. Given that EE may influence cue salience and reward valuation, it will be important to test EE in isolation with other relapse modalities. Second, while corticosterone provides a meaningful measure of HPA axis activity, other neurobiological readouts of stress system function like CRF expression or noradrenergic activity may clarify more detailed underlying mechanisms. Past research has demonstrated the sufficiency of corticosterone and necessity of glucocorticoid receptor activation in stress and context induced reinstatement, respectfully (Erb et al., 1998; Stringfield et al., 2016), but these are coupled with reports indicating the requisite role of norepinephrine (Mantsch et al., 2010) and noradrenergic activity from locus ceruleus (Markovic et al., 2024) in the reinstatement of drug-seeking behavior. Here, we use corticosterone concentrations as a proxy for HPA axis activity, but the observed effects on drug-seeking behavior are likely a culmination of several signaling cascades recruited upon HPA axis activation. Therefore, it will be important for future studies to further dissect potential stress mechanisms impacted by environmental enrichment and opioid use. Finally, although singly housed enrichment allowed isolation of object-based effects, social enrichment remains a powerful determinant of addiction vulnerability, and future work should directly compare object-only, social-only, and combined enrichment approaches.

### Conclusions

Our findings show that long-term, object-based environmental enrichment is sufficient to blunt fentanyl self-administration, reduce the persistence of drug-seeking, and protect against stress-induced reinstatement and stress reactivity. These results highlight the power of environmental context—and specifically, access to stimulating, engaging, and rewarding nonsocial activities—to alter opioid abuse vulnerability. In the context of the ongoing fentanyl crisis, these data underscore the potential translational utility of enrichment-inspired interventions that are feasible in socially isolated or resource-limited settings. Such interventions may provide a low-risk and scalable means to reduce opioid use and relapse risk, complementing existing pharmacological and behavioral therapies.

## Synthesis

Reviewing Editor: Sam Golden, The University of Washington Decisions are customarily a result of the Reviewing Editor and the peer reviewers coming together and discussing their recommendations until a consensus is reached. When revisions are invited, a fact-based synthesis statement explaining their decision and outlining what is needed to prepare a revision will be listed below. The following reviewer(s) agreed to reveal their identity: Anushree Karkhanis.

Dear Dr. Moron,

Your manuscript has been reviewed by two experts in the field, who both found it to be of high interest and merit. The authors have some suggestions that would further strengthen the manuscript prior to publication. We also specifically had a discussion related to Reviewer 1 comment 6, about the potential approach of splitting rats into high and low responder groups. Ultimately, we will leave this decision to you and the authors, but ask in your rebuttal that you provide a rationale for the decision. Below, please find the reviewers full comments. I look forward to reviewing a revised manuscript.

Reviewer 1:

In this manuscript, the authors investigate the effects of environmental enrichment on fentanyl self-administration and associated alterations in corticosterone levels. The findings demonstrate that long-term environmental enrichment reduces both fentanyl intake and basal corticosterone concentrations. In addition, exposure to a pharmacological stressor, such as yohimbine, elicits heightened HPA axis responsivity, as evidenced by yohimbine-induced elevations in corticosterone levels. Overall, the results are compelling, and the experimental approach appears sound. However, several concerns outlined below should be addressed.

1) Please clarify the number of animals that were bred in-house versus those procured from external vendors. Additionally, were there any differences in corticosterone levels or behavioral outcomes between these two groups? Prior studies have reported significant differences between procured and in-house-bred rats with respect to anxiety-like behaviors and ethanol consumption (e.g., PMID: 22924742), which could be a potential confounding factor in the present study.

2) The authors report a wide range of body weights for the animals used. While this variability may in part reflect sex differences, it would be important to also report the ages of the animals. Social isolation during adolescence has been shown to alter drug self-administration, and therefore developmental stage represents a critical factor that should be considered and clarified in the present study.

3) The duration of housing enrichment exposure was relatively brief (3-5 days). This limited exposure should be explicitly stated throughout the manuscript, as it is for the corticosterone data. Without this clarification, the data presented in Figure 2A may be overinterpreted with respect to the effects of environmental enrichment.

4) For the within-group analyses presented in Figure 3, sessions 2-5 should be compared to session 1, rather than comparing sessions 1-4 to session 5. Behavioral responses during sessions 2-5 are likely influenced by prior session experience, and using session 1 as the reference point would provide a more appropriate and interpretable baseline for these comparisons.

5) While the other behavioral measures are analyzed using three-way ANOVAs to assess potential sex differences, the corticosterone data are not analyzed for sex effects at any point in the study. Prior work has demonstrated sex differences in anxiety-like behaviors following manipulations of housing conditions. Moreover, although short-term environmental enrichment appears not to alter corticosterone levels overall, the data suggest potential sex differences in corticosterone responses following fentanyl self-administration. Accordingly, inclusion of sex as a factor in the corticosterone analyses would strengthen the rigor and interpretability of the findings.

6) The variability in active lever responding depicted in Figure 4A appears relatively high. Were the groups subdivided into high- versus low-responders for analysis? Additionally, could this variability be driven by individual differences in corticosterone levels or stress responsivity? Clarification or exploratory analyses addressing these possibilities would help to contextualize the observed behavioral variability.

7) Figure 5: Given the presence of baseline group differences (NE vs. EE) prior to extinction, it would be important to normalize the extinction data, for example by calculating the percentage reduction in responding using Day 1 of extinction as the baseline. This approach would allow for a more appropriate comparison of extinction-related changes between groups. The corresponding discussion should also be revised to reflect the outcomes of the normalized analyses.

8) The use of yohimbine as a pharmacological stressor is appropriate; however, given its known interactions with the noradrenergic system, the authors should include a caveat in the Discussion addressing this mechanism. Specifically, it would be important to consider and discuss the potential contribution of norepinephrine signaling to fentanyl self-administration and how this may influence the interpretation of the stress-related effects observed.

9) Although a minor point, it is important to clarify that references 48 and 50 do not report changes in mesolimbic dopamine. Rather, these studies identify alterations in the mesocortical dopamine system. The manuscript should be corrected to accurately reflect these findings.

10) Minor: typo in line 299 - "that" should be "than"

Reviewer 2:

The manuscript builds upon existing literature demonstrating enrichment decreases drug-related behavior. The authors ask how environmental enrichment influences IV fentanyl self-administration, extinction, and pharmacologic-stress-induced relapse behavior in rats. They show environmental enrichment decreases fentanyl intake, concomitant with trending decreases in corticosterone, and slightly accelerates extinction. Enriched rats show reduced relapse-like behavior following yohimbine, related to blunted HPA-axis activation relative to non-enriched rats. The manuscript is very straightforward, the findings are clear, and they do an excellent job considering sex as a biological variable. There are only a few minor concerns.

1- Accelerated extinction and reduced reinstatement in enriched rats may be attributed to their reduced fentanyl intake. Statements like, "enrichment attenuates stress-induced reinstatement" (line 311) assume causation between enrichment and stress-induced reinstatement that is not necessarily supported by the data shown. A group that receives enrichment after self-administering similar amounts of fentanyl would better support this conclusion. I suggest instead, that the authors should simply state the findings (e.g., "EE rats exhibit attenuated stress-induced reinstatement) instead of attributing behavior to enrichment.

2- Line 279 states "EE suppressed fentanyl self-administration during both acquisition and maintenance." However, the EE rats only had reduced lever presses compared to NE rats on one day, both groups discriminate between active and inactive levers, and there don't appear to be differences in overall intake during the "acquisition" phase. Please remove the reference to "acquisition" in this line.
