# Supplementary material for: Environmental Enrichment Attenuates Fentanyl-Seeking Behavior and Protects against Stress-Induced Reinstatement in Both Male and Female Rats
Source: eNeuro. 2026 Apr 16;13(4):ENEURO.0447-25.2026. doi: 10.1523/ENEURO.0447-25.2026 (PMC13095401; doi:10.1523/ENEURO.0447-25.2026)
Supplement: Figure 2-1 — Table with statistical reporting for Figure 2. Download Figure 2-1, DOCX file. [file eneuro-13-ENEURO.0447-25.2026-s003.docx]

Figure 2-1. Table with Statistical Reporting for Figure 2

| **Figure** | **Data Analyzed** | **Primary**  **Analysis** | **Post-Hoc**  **Analysis** | **Comparison** | **P value** | **Statistic** |
| --- | --- | --- | --- | --- | --- | --- |
|  |  | Unpaired T-test |  | NE vs. EE | 0.4160 | t=0.8231, df=35 |
| **2B** | Pre-SA |  |  | Sex x Enrichment | 0.8000 | F (1, 33) = 0.06523 |
|  | Corticosterone | 2-way ANOVA |  | Sex | 0.9965 | F (1, 33) = 1.907e-005 |
|  |  |  |  | Enrichment | 0.4203 | F (1, 33) = 0.6660 |
