# Supplementary material for: Environmental Enrichment Attenuates Fentanyl-Seeking Behavior and Protects against Stress-Induced Reinstatement in Both Male and Female Rats
Source: eNeuro. 2026 Apr 16;13(4):ENEURO.0447-25.2026. doi: 10.1523/ENEURO.0447-25.2026 (PMC13095401; doi:10.1523/ENEURO.0447-25.2026)
Supplement: Figure 3-1 — Table with statistical reporting for Figure 3. Download Figure 3-1, DOCX file. [file eneuro-13-ENEURO.0447-25.2026-s002.docx]

Figure 3-1. Statistical Reporting for Figure 3

| **Figure** | **Data Analyzed** | **Primary Analysis** | **Post-Hoc Analysis** | **Comparison** | **P value** | **Statistic** |
| --- | --- | --- | --- | --- | --- | --- |
|  |  |  |  | Session | <0.0001 | F (4, 132) = 6.555 |
|  |  |  |  | (Enrichment) | 0.2195 | F (1, 33) = 1.567 |
|  | Acquisition: Active Lever Presses | RM 3-way ANOVA |  | Sex Session x (Enrichment)  Session x Sex Enrichment x Sex  Session x Enrichment x Sex | 0.4020  0.1234  0.8533  0.1608  0.0427 | F (1, 33) = 0.7207  F (4, 132) = 1.848  F (4, 132) = 0.3361  F (1, 33) = 2.058  F (4, 132) = 2.541 |
|  |  |  |  | Session x Enrichment | 0.1444 | F (4, 64) = 3.683 |
|  |  | RM 2-way |  | Session | <0.0001 | F (4, 64) = 2.911 |
|  |  | ANOVA |  | Enrichment | 0.1991 | F (1, 16) = 0.01351 |
|  |  |  |  | Subject | <0.0001 | F (16, 64) = 5.060 |
|  |  |  |  | Session | 0.0463 | F (4, 72) = 2.551 |
|  |  |  |  | Lever (Active vs Inactive) | <0.0001 | F (1, 18) = 37.31 |
|  |  |  |  | Session x Lever | 0.0006 | F (4, 57) = 5.677 |
| **3A** | Acquisition: NE Lever Discrimination | RM 2-way ANOVA | Šídák's multiple | ***NE Active vs. Inactive Lever***  Session 1  Session 2 |  |  |
|  |  |  |  |  | 0.2617  0.0341 |  |
|  |  |  | comparisons t | Session 3 | 0.0003 |  |
|  |  |  | est | Session 4 | 0.0002 |  |
|  |  |  |  | Session 5 | <0.0001 |  |
|  |  |  |  | Session | 0.2653 | F (4, 84) = 1.331 |
|  |  |  |  | Lever (Active vs Inactive) | <0.0001 | F (1, 21) = 34.53 |
|  |  |  |  | Session x Lever | 0.5644 | F (4, 64) = 0.7458 |
|  | Acquisition: EE Lever Discrimination | RM 2-way ANOVA | Šídák's multiple | ***EE Active vs. Inactive Lever***  Session 1  Session 2 |  |  |
|  |  |  |  |  | 0.0649  0.0265 |  |
|  |  |  | comparisons t | Session 3 | 0.0009 |  |
|  |  |  | est | Session 4 | 0.0001 |  |
|  |  |  |  | Session 5 | 0.0076 |  |
|  |  |  |  | Session x Enrichment | 0.0092 | F (4, 64) = 3.683 |
|  |  |  |  | Session | 0.0282 | F (4, 64) = 2.911 |
|  |  |  |  | Enrichment | 0.9089 | F (1, 16) = 0.01351 |
|  |  |  |  | Subject | <0.0001 | F (16, 64) = 5.060 |
|  |  |  |  | ***NE Male vs EE Male*** |  |  |
|  |  |  |  | Session 1 | 0.8687 |  |
|  |  |  |  | Session 2 | 0.4506 |  |
|  |  |  |  | Session 3 | 0.1275 |  |
|  |  |  |  | Session 4 | 0.6037 |  |
|  |  |  |  | Session 5 | 0.0293 |  |
|  |  |  |  | ***NE Male Sessions*** |  |  |
|  |  |  |  | 1 vs 2 | 0.9650 |  |
|  |  |  |  | 1 vs 3 | 0.9276 |  |
|  |  |  |  | 1 vs 4 | >0.9999 |  |
|  |  |  |  | 1 vs 5 | 0.0163 |  |
| **3B** | Acquisition: Male  Active Lever Presses | RM 2-way  ANOVA | Tukey's | 2 vs 3  2 vs 4 | 0.9999  0.9544 |  |
|  |  |  | Multiple | 2 vs 5 | 0.0022 |  |
|  |  |  | Comparison's | 3 vs 4 | 0.9113 |  |
|  |  |  | Test | 3 vs 5 | 0.0014 |  |
|  |  |  |  | 4 vs 5 | 0.0188 |  |

|  |  |  |  | ***EE Male Sessions*** |  |  |
| --- | --- | --- | --- | --- | --- | --- |
|  |  |  |  | 1 vs 2 | 0.9767 |  |
|  |  |  |  | 1 vs 3 | 0.5450 |  |
|  |  |  |  | 1 vs 4 | 0.8371 |  |
|  |  |  |  | 1 vs 5 | 0.9608 |  |
|  |  |  |  | 2 vs 3 | 0.8773 |  |
|  |  |  |  | 2 vs 4 | 0.9913 |  |
|  |  |  |  | 2 vs 5 | >0.9999 |  |
|  |  |  |  | 3 vs 4 | 0.9875 |  |
|  |  |  |  | 3 vs 5 | 0.9114 |  |
|  |  |  |  | 4 vs 5 | 0.9963 |  |
|  |  |  |  | Session x Enrichment | 0.5070 | F (4, 68) = 0.8361 |
| **3C** | Acquisition: Female  Active Lever Presses | RM 2-way  ANOVA |  | Session  Enrichment | 0.0059  0.0467 | F (4, 68) = 3.970  F (1, 17) = 4.602 |
|  |  |  |  | Subject | 0.0008 | F (17, 68) = 2.959 |
|  |  |  |  | Session | <0.0001 | F (4, 132) = 6.555 |
|  |  |  |  | (Enrichment) | 0.5720 | F (1, 33) = 1.567 |
| **3D** | Acquisition: Fentanyl Intake | RM 3-way ANOVA |  | Sex Session x (Enrichment)  Session x Sex Enrichment x Sex  Session x Enrichment x Sex | 0.5325  0.4187  0.9039  0.0710  0.0043 | F (1, 33) = 0.7207  F (4, 132) = 1.848  F (4, 132) = 0.3361  F (1, 33) = 2.058  F (4, 132) = 2.541 |
|  |  |  |  | Session x Enrichment | 0.4922 | F (4, 64) = 3.683 |
|  |  | RM 2-way |  | Session | <0.0001 | F (4, 64) = 2.911 |
|  |  | ANOVA |  | Enrichment | 0.5331 | F (1, 16) = 0.01351 |
|  |  |  |  | Subject | <0.0001 | F (16, 64) = 5.060 |
|  |  |  |  | Session x Enrichment | 0.0061 | F (4, 64) = 3.974 |
|  |  |  |  | Session | 0.0018 | F (4, 64) = 4.821 |
|  |  |  |  | Enrichment | 0.4642 | F (1, 16) = 0.5624 |
|  |  |  |  | Subject | <0.0001 | F (16, 64) = 6.408 |
|  |  |  |  | ***NE Male vs EE Male*** |  |  |
|  |  |  |  | Session 1 | 0.5220 |  |
|  |  |  |  | Session 2 | 0.2266 |  |
|  |  |  |  | Session 3 | 0.0297 |  |
|  |  |  |  | Session 4 | 0.6788 |  |
|  |  |  |  | Session 5 | 0.1255 |  |
|  |  |  |  | ***NE Male Sessions*** |  |  |
|  |  |  |  | 1 vs 2 | 0.9918 |  |
|  |  |  |  | 1 vs 3 | >0.9999 |  |
|  |  |  |  | 1 vs 4 | 0.7211 |  |
|  |  |  |  | 1 vs 5 | 0.0015 |  |
| **3E** | Acquisition: Male  Fentanyl Intake | RM 2-way  ANOVA | Tukey's | 2 vs 3  2 vs 4 | 0.9890  0.4440 |  |
|  |  |  | Multiple | 2 vs 5 | 0.0003 |  |
|  |  |  | Comparison's | 3 vs 4 | 0.7420 |  |
|  |  |  | Test | 3 vs 5 | 0.0017 |  |
|  |  |  |  | 4 vs 5 | 0.0584 |  |
|  |  |  |  | ***EE Male Sessions*** |  |  |
|  |  |  |  | 1 vs 2 | 0.9953 |  |
|  |  |  |  | 1 vs 3 | 0.1173 |  |
|  |  |  |  | 1 vs 4 | 0.8303 |  |
|  |  |  |  | 1 vs 5 | 0.7985 |  |
|  |  |  |  | 2 vs 3 | 0.2509 |  |
|  |  |  |  | 2 vs 4 | 0.9627 |  |

|  |  |  |  | 1. vs 5 2. vs 4 3. vs 5 4. vs 5 | 0.9488  0.6354  0.6746  >0.9999 |  |
| --- | --- | --- | --- | --- | --- | --- |
| **3F** | Acquisition: Female Fentanyl Intake | RM 2-way ANOVA |  | Session x enrichment  Session enrichment  Subject | 0.4822  <0.0001  0.0346  0.0025 | F (4, 68) = 0.8773  F (4, 68) = 8.981  F (1, 17) = 5.278  F (17, 68) = 2.633 |
| **3G** | Acquisition: Cort | 2-way ANOVA |  | Sex x Enrichment  Sex Enrichment | 0.6608  0.2189  0.0776 | F (1, 34) = 0.1959  F (1, 34) = 1.569  F (1, 34) = 3.311 |
| **3H** | Cort vs Active Lever | Linear Reg. |  | Cort vs. Active Lever Presses | 0.7730 | r^2^=0.002340 |
