# Supplementary material for: Environmental Enrichment Attenuates Fentanyl-Seeking Behavior and Protects against Stress-Induced Reinstatement in Both Male and Female Rats
Source: eNeuro. 2026 Apr 16;13(4):ENEURO.0447-25.2026. doi: 10.1523/ENEURO.0447-25.2026 (PMC13095401; doi:10.1523/ENEURO.0447-25.2026)
Supplement: Figure 4-1 — Table with statistical reporting for Figure 4. Download Figure 4-1, DOCX file. [file eneuro-13-ENEURO.0447-25.2026-s004.docx]

Figure 4-1. Statistical Reporting for Figure 4

| **Figure** | **Data Analyzed** | **Primary Analysis** | **Post-Hoc Analysis** | **Comparison** | **P value** | **Statistic** |
| --- | --- | --- | --- | --- | --- | --- |
| **4A** | Maintenance: Active Lever Responding | RM 3-way ANOVA |  | Session  Enrichment  Sex Session x Enrichment  Session x Sex Enrichment x Sex  Session x Enrichment x Sex | 0.0025  0.0472  0.6596  0.2715  0.1060  0.3710  0.1966 | F (9, 297) = 2.912  F (1, 33) = 4.249  F (1, 33) = 0.1976  F (9, 297) = 1.238  F (9, 297) = 1.630  F (1, 33) = 0.8226  F (9, 297) = 1.380 |
|  |  | RM 2-way ANOVA |  | Session x Enrichment  Session Enrichment  Subject | 0.2151  0.0019  0.0398  <0.0001 | F (9, 315) = 1.340  F (9, 315) = 3.004  F (1, 35) = 4.558  F (35, 315) = 27.56 |
| **4B** | Maintenance: Fentanyl Intake | RM 3-way ANOVA |  | Session  Enrichment  Sex Session x Enrichment  Session x Sex Enrichment x Sex  Session x Enrichment x Sex | 0.0023  0.0222  0.9810  0.4222  0.3923  0.5207  0.9481 | F (9, 297) = 2.952  F (1, 33) = 5.756  F (1, 33) = 0.0005735  F (9, 297) = 1.022  F (9, 297) = 1.060  F (1, 33) = 0.4214  F (9, 297) = 0.3716 |
|  |  | RM 2-way ANOVA |  | Session x Enrichment  Session Enrichment  Subject | 0.7880  0.0035  0.0297  <0.0001 | F (9, 315) = 0.6106  F (9, 315) = 2.809  F (1, 35) = 5.139  F (35, 315) = 20.45 |
| **4C** | Maintenance: Cort | 2-way ANOVA |  | Sex x Enrichment  Sex Enrichment | 0.7286  0.0010  0.0683 | F (1, 34) = 0.1224  F (1, 34) = 13.05  F (1, 34) = 3.544 |
| **4D** | Cort vs Active Lever | Linear Reg. |  | Cort vs. Active Lever Presses | 0.7907 | r^2^=0.001982 |
