# Supplementary material for: Environmental Enrichment Attenuates Fentanyl-Seeking Behavior and Protects against Stress-Induced Reinstatement in Both Male and Female Rats
Source: eNeuro. 2026 Apr 16;13(4):ENEURO.0447-25.2026. doi: 10.1523/ENEURO.0447-25.2026 (PMC13095401; doi:10.1523/ENEURO.0447-25.2026)
Supplement: Figure 5-1 — Table with statistical reporting for Figure 5. Download Figure 5-1, DOCX file. [file eneuro-13-ENEURO.0447-25.2026-s005.docx]

Figure 5-1. Table with Statistical Reporting for Figure 5

| **Figure** | **Data Analyzed** | **Primary**  **Analysis** | **Post-Hoc**  **Analysis** | **Comparison** | **P value** | **Statistic** |
| --- | --- | --- | --- | --- | --- | --- |
|  |  |  |  | Session | <0.0001 | F (6, 204) = 14.45 |
|  |  |  |  | Enrichment | 0.1618 | F (1, 34) = 2.045 |
|  |  | RM 3-way ANOVA |  | Sex Session x Enrichment  Session x Sex | 0.1021  0.0494  0.2016 | F (1, 34) = 2.824  F (6, 204) = 2.149  F (6, 204) = 1.438 |
|  |  |  |  | Enrichment x Sex | 0.7356 | F (1, 34) = 0.1159 |
|  |  |  |  | Session x Enrichment x Sex | 0.9741 | F (6, 204) = 0.2077 |
|  |  |  |  | Session x Enrichment | 0.0504 | F (6, 216) = 2.137 |
|  |  |  |  | Session | <0.0001 | F (6, 216) = 14.55 |
|  |  |  |  | Enrichment | 0.1453 | F (1, 36) = 2.216 |
|  |  |  |  | Subject | <0.0001 | F (36, 216) = 5.000 |
|  |  |  |  | ***Extinction Active Lever: NE*** |  |  |
|  |  |  |  | Session 1 | 0.0823 |  |
|  |  |  |  | Session 2 | 0.0021 |  |
|  |  |  |  | Session 3 | 0.2932 |  |
|  |  |  |  | Session 4 | 0.4299 |  |
|  |  |  |  | Session 5 | 0.4183 |  |
|  |  |  |  | Session 6 | 0.0908 |  |
|  |  |  |  | Session 7 | 0.7177 |  |
|  |  |  |  | ***NE Exctinction Responding*** |  |  |
|  |  |  |  | Session 1 vs. Session 2 | >0.9999 |  |
|  |  |  |  | Session 1 vs. Session 3 | 0.0010 |  |
|  |  |  |  | Session 1 vs. Session 4 | 0.0013 |  |
|  |  |  |  | Session 1 vs. Session 5 | <0.0001 |  |
|  |  |  |  | Session 1 vs. Session 6 | <0.0001 |  |
|  |  |  |  | Session 1 vs. Session 7 | <0.0001 |  |
|  |  |  |  | Session 2 vs. Session 3 | 0.0224 |  |
|  |  |  |  | Session 2 vs. Session 4 | 0.0262 |  |
|  |  |  |  | Session 2 vs. Session 5 | 0.0014 |  |
|  |  |  |  | Session 2 vs. Session 6 | <0.0001 |  |
| **5A** | Extinction: Active Lever Responding |  |  | Session 2 vs. Session 7  Session 3 vs. Session 4  Session 3 vs. Session 5 | <0.0001  >0.9999  >0.9999 |  |
|  |  |  |  | Session 3 vs. Session 6 | 0.6384 |  |
|  |  | RM 2-way |  | Session 3 vs. Session 7 | 0.4848 |  |
|  |  | ANOVA | Šídák's | Session 4 vs. Session 5 | >0.9999 |  |
|  |  |  | multiple | Session 4 vs. Session 6 | 0.5973 |  |
|  |  |  | comparisons | Session 4 vs. Session 7 | 0.4454 |  |
|  |  |  | test | Session 5 vs. Session 6 | 0.9942 |  |
|  |  |  |  | Session 5 vs. Session 7 | 0.9740 |  |
|  |  |  |  | Session 6 vs. Session 7 | >0.9999 |  |
|  |  |  |  | ***EE Extinction Responding*** |  |  |
|  |  |  |  | Session 1 vs. Session 2 | 0.1787 |  |
|  |  |  |  | Session 1 vs. Session 3 | 0.0129 |  |
|  |  |  |  | Session 1 vs. Session 4 | 0.0468 |  |

|  |  |  |  | Session 1 vs. Session 5  Session 1 vs. Session 6  Session 1 vs. Session 7  Session 2 vs. Session 3  Session 2 vs. Session 4  Session 2 vs. Session 5  Session 2 vs. Session 6  Session 2 vs. Session 7  Session 3 vs. Session 4  Session 3 vs. Session 5  Session 3 vs. Session 6  Session 3 vs. Session 7  Session 4 vs. Session 5  Session 4 vs. Session 6  Session 4 vs. Session 7  Session 5 vs. Session 6  Session 5 vs. Session 7  Session 6 vs. Session 7 | 0.0021  0.0014  0.0023  >0.9999  >0.9999  0.9852  0.9658  0.9878  >0.9999  >0.9999  >0.9999  >0.9999  >0.9999  0.9997  >0.9999  >0.9999  >0.9999  >0.9999 |  |
| --- | --- | --- | --- | --- | --- | --- |
| **5B** | Extinction: Cort | 2-way ANOVA |  | Sex x Enrichment  Sex Enrichment | 0.3424  0.3759  0.1178 | F (1, 33) = 0.9281  F (1, 33) = 0.8056  F (1, 33) = 2.579 |
| **5C** | Cort vs Active Lever | Linear Reg. |  | Cort vs. Active Lever Presses | 0.4606 | r^2^=0.01661 |
