# Supplementary material for: Environmental Enrichment Attenuates Fentanyl-Seeking Behavior and Protects against Stress-Induced Reinstatement in Both Male and Female Rats
Source: eNeuro. 2026 Apr 16;13(4):ENEURO.0447-25.2026. doi: 10.1523/ENEURO.0447-25.2026 (PMC13095401; doi:10.1523/ENEURO.0447-25.2026)
Supplement: Figure 6-1 — Table with statistical reporting for Figure 6. Download Figure 6-1, DOCX file. [file eneuro-13-ENEURO.0447-25.2026-s006.docx]

Figure 6-1. Statistical Reporting for Figure 6

| **Figure** | **Data Analyzed** | **Primary Analysis** | **Post-Hoc Analysis** | **Comparison** | **P value** | **Statistic** |
| --- | --- | --- | --- | --- | --- | --- |
|  |  |  |  | Session | <0.0001 | F (1, 34) = 38.11 |
|  |  |  |  | Enrichment | 0.0257 | F (1, 34) = 5.440 |
|  |  |  |  | Sex | 0.7014 | F (1, 34) = 0.1495 |
|  |  | RM 3-way ANOVA |  | Session x Enrichment | 0.0211 | F (1, 34) = 5.844 |
|  |  |  |  | Session x Sex | 0.7334 | F (1, 34) = 0.1179 |
|  |  |  |  | Enrichment x Sex | 0.9150 | F (1, 34) = 0.01155 |
|  |  |  |  | Session x Enrichment x Sex | 0.9701 | F (1, 34) = 0.001427 |
| **6A** | Extinction vs Reinstatement Active Lever Responding |  |  |  |  |  |
|  |  |  |  | Session x Enrichment  Session Enrichment | 0.0166  <0.0001  0.0202 | F (1, 36) = 6.316  F (1, 36) = 40.75  F (1, 36) = 5.908 |
|  |  |  |  | Subject | 0.3417 | F (36, 36) = 1.147 |
|  |  | RM 2-way ANOVA |  | ***Active Lever Presses*** Last Day Extinction: NE vs EE Reinstatement: NE vs EE  NE: Extinction vs Reinstatement EE: Extinction vs Reinstatement |  |  |
|  |  |  | Šídák's multiple comparisons test |  |  |  |
|  |  |  |  |  | 0.9976  0.0016  <0.0001  0.0158 |  |
|  |  |  |  | Sex x Enrichment | 0.0028 | F (1, 33) = 10.44 |
|  |  |  |  | Sex | 0.0149 | F (1, 33) = 6.599 |
|  |  |  |  | Enrichment | 0.0380 | F (1, 33) = 4.674 |
| **6B** | Reinstatement Cort | 2-way ANOVA | Šídák's | Males: EE vs. NE | 0.7104 |  |
|  |  |  | multiple | Females: EE vs. NE | 0.0010 |  |
|  |  |  | comparisons | EE: Males vs. Females | 0.8683 |  |
|  |  |  | test | NE: Males vs. Females | 0.0006 |  |
| **6C** | Cort vs Active Lever | Simple Linear Reg. |  | Cort vs. Active Lever Presses | 0.0075 | r^2^=0.1872 |
|  |  |  |  | Session | <0.0001 | F (4, 133) = 12.69 |
|  |  |  |  | Sex | 0.0033 | F (1, 34) = 9.998 |
|  |  |  |  | Enrichment | 0.0030 | F (1, 34) = 10.21 |
|  |  | RM 3-way ANOVA |  | Session x Sex | 0.7503 | F (4, 133) = 0.4801 |
| **6D** | Corticosterone by  training phase |  |  | Session x Enrichment  Enrichment x Sex | 0.3899  0.2145 | F (4, 133) = 1.038  F (1, 34) = 1.600 |
|  |  |  |  | Session x Enrichment x Sex | 0.0753 | F (4, 133) = 2.174 |
|  |  |  |  | Session | <0.0001 | F (4, 141) = 12.99 |
|  |  | RM 2-way ANOVA |  | Enrichment | 0.0046 | F (1, 36) = 9.142 |
|  |  |  |  | Session x Enrichment | 0.7071 | F (4, 141) = 0.5393 |
|  |  |  |  | Session x Enrichment | 0.3022 | F (4, 63) = 5.852 |
| **6E** | Male Cort by Phase | RM 2-way ANOVA |  | Session | 0.0019 | F (1, 16) = 1.726 |
|  |  |  |  | Enrichment | 0.1683 | F (4, 63) = 1.190 |
| **6F** | Female Cort by Phase | RM 2-way ANOVA |  | Session x Enrichment  Session Enrichment | 0.1182  <0.0001  0.0085 | F (4, 64) = 1.918  F (4, 64) = 8.053  F (1, 16) = 9.000 |
